# *Thermus oshimai* JL-2 and *T. thermophilus* JL-18 genome analysis illuminates pathways for carbon, nitrogen, and sulfur cycling

**DOI:** 10.4056/sigs.3667269

**Published:** 2013-02-25

**Authors:** Senthil K. Murugapiran, Marcel Huntemann, Chia-Lin Wei, James Han, J. C. Detter, Cliff Han, Tracy H. Erkkila, Hazuki Teshima, Amy Chen, Nikos Kyrpides, Konstantinos Mavrommatis, Victor Markowitz, Ernest Szeto, Natalia Ivanova, Ioanna Pagani, Amrita Pati, Lynne Goodwin, Lin Peters, Sam Pitluck, Jenny Lam, Austin I. McDonald, Jeremy A. Dodsworth, Tanja Woyke, Brian P. Hedlund

**Affiliations:** 1 School of Life Sciences, University of Nevada Las Vegas, Las Vegas, NV, USA; 2 Department of Energy Joint Genome Institute, Walnut Creek, CA, USA; 3 Los Alamos National Laboratory, Los Alamos, NM, USA

**Keywords:** *Thermus*, *Thermus oshimai*, *Thermus thermophilus*, thermophiles, hot springs, denitrification, nitrous oxide, Great Basin

## Abstract

The complete genomes of *Thermus oshimai* JL-2 and *T. thermophilus* JL-18 each consist of a circular chromosome, 2.07 Mb and 1.9 Mb, respectively, and two plasmids ranging from 0.27 Mb to 57.2 kb. Comparison of the *T. thermophilus* JL-18 chromosome with those from other strains of *T. thermophilus* revealed a high degree of synteny, whereas the megaplasmids from the same strains were highly plastic. The *T. oshimai* JL-2 chromosome and megaplasmids shared little or no synteny with other sequenced *Thermus* strains. Phylogenomic analyses using a concatenated set of conserved proteins confirmed the phylogenetic and taxonomic assignments based on 16S rRNA phylogenetics. Both chromosomes encode a complete glycolysis, tricarboxylic acid (TCA) cycle, and pentose phosphate pathway plus glucosidases, glycosidases, proteases, and peptidases, highlighting highly versatile heterotrophic capabilities. Megaplasmids of both strains contained a gene cluster encoding enzymes predicted to catalyze the sequential reduction of nitrate to nitrous oxide; however, the nitrous oxide reductase required for the terminal step in denitrification was absent, consistent with their incomplete denitrification phenotypes. A *sox* gene cluster was identified in both chromosomes, suggesting a mode of chemolithotrophy. In addition, *nrf* and *psr* gene clusters in *T. oshmai* JL-2 suggest respiratory nitrite ammonification and polysulfide reduction as possible modes of anaerobic respiration.

## Introduction

The Great Boiling Spring (GBS) geothermal system is located in the northwestern Great Basin near the town of Gerlach, Nevada. Geothermal activity is driven by deep circulation of meteoric water, which rises along range-front faults at temperatures up to 96 ºC. A considerable volume of geomicrobiology research has been conducted in the GBS system, including coordinated cultivation-independent microbiology and geochemistry studies [1-4], habitat niche modeling [3], thermodynamic modeling [1,5], microbial cultivation and physiology [6,7], and integrated studies of the nitrogen biogeochemical cycle (N-cycle [5,6,8]). The latter group of studies is arguably the most detailed body of work on the N-cycle in any geothermal system. Those studies revealed a dissimilatory N-cycle based on oxidation and subsequent denitrification of ammonia supplied in the geothermal source water.

In high temperature sources such as GBS and Sandy’s Spring West (SSW), ammonia oxidation occurs at temperatures up to at least 82 ºC at rates comparable to those in nonthermal aquatic sediments [5]. Several lines of evidence, including deep 16S rRNA gene pyrosequencing datasets and quantitative PCR, suggest ammonia oxidation is carried out by a single species of ammonia-oxidizing archaea closely related to “*Candidatus* Nitrosocaldus yellowstonii”, which comprises a substantial proportion of the sediment microbial community in some parts of the springs [5,9]. Nitrite oxidation appears to be sluggish or non-existent in the high temperature source pools since nitrite accumulates in these systems and 16S rRNA gene sequences for nitrite-oxidizing bacteria have not been detected in clone library and pyrotag censuses [1,5]. Finally, the nitrite and nitrate that are produced are denitrified in the sediments to both nitrous oxide and dinitrogen; however, a high flux of nitrous oxide, particularly in the ~80 ºC source pool of GBS, suggested the importance of incomplete denitrifiers [6] and electron donor stimulation experiments suggested a key role for heterotrophic denitrifiers [5].

A subsequent cultivation study of heterotrophic denitrifiers in GBS and SSW resulted in the isolation of a large number of denitrifiers belonging to *Thermus thermophilus* and *T. oshimai*, including strains *T. oshimai* JL-2 and *T. thermophilus* JL-18 [6]. Strikingly, although *Thermus* strains were isolated using four different isolation strategies, nine different electron donor/acceptor combinations, and four different sampling dates, all isolates of these two species were able to convert nitrate-N stoichiometrically to nitrous oxide-N, but appeared unable to reduce nitrous oxide to dinitrogen. This physiology, combined with high nitrous oxide fluxes *in situ* suggested a significant role of *T. oshimai* and *T. thermophilus* in the unusual N-cycle in these hot springs. However, the genetic basis of this phenotype remained unknown. Here we present the complete genome sequences of *T. oshimai* JL-2 and *T. thermophilus* JL-18, compare them to genomes of other sequenced *Thermus* spp., and discuss them within the context of their potential impacts on biogeochemical cycling of carbon, nitrogen, sulfur, and iron.

## Classification and features

**Figure 1:**
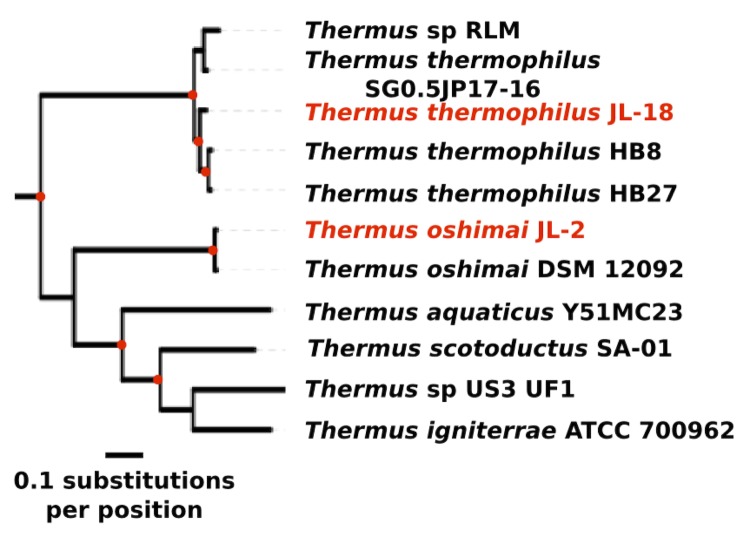
Phylogenomic tree highlighting the position of *Thermus oshimai* JL-2 and *Thermus thermophilus* JL-18. Thirty-one bacterial phylogenetic markers were identified using Amphora [10]. Maximum-likelihood analysis was carried out with a concatenated alignment of all 31 proteins using RAxML Version 7.2.6 [11] and the tree was visualized using iTOL [12]. Red circles indicate bootstrap support >80% (100 replicates). Scale bar indicates 0.1 substitutions per position. The protein FASTA files for all the species are from NCBI, except for the following species, which are from IMG: *Thermus igniterrae* ATCC 700962 (Taxon OID: 2515935625), *Thermus oshimai* DSM 12092 (Taxon OID: 2515463139), *Thermus oshimai* JL-2 (Taxon OID: 2508706991), *Thermus sp.* RLM (Taxon OID: 2514335427).

The genus *Thermus* currently comprises 16 species and includes the well-known *T. aquaticus* and the genetically tractable *T. thermophilus*. The genome of *T. oshimai* JL-2 is the first finished genome to be reported from that species, while *T. thermophilus* JL-18 is the fourth genome to be sequenced from that species, the other being *T. thermophilus* HB27, HB8, and SG0.5JP17-16. Figure 1 shows the relationship of *T. oshimai* JL-2 and *T. thermophilus* JL-18 to other *Thermus* species, as determined by phylogenomic analysis of highly conserved genes, which supports the taxonomic identities previously determined by 16S rRNA gene phylogenetic analysis [6]. Table 1 shows general features of *T. oshimai* JL-2 and *T. thermophilus* JL-18.

**Table 1(a):** Classification and general features of *Thermus oshimai* JL-2 according to the MIGS recommendations [13].

**MIGS ID**	**Property**	**Term**	**Evidence code**^a^
	Current classification	Domain *Bacteria*	TAS [14]
		Phylum *Deinococcus-Thermus*	TAS [15]
		Class *Deinococci*	TAS [16,17]
		Order *Thermales*	TAS [16,18]
		Family *Thermaceae*	TAS [16,19]
		Genus *Thermus*	TAS [20-22]
		Species *Thermus oshimai*	TAS [23]
		Type strain JL-2	TAS [6]
	Gram stain	Negative	TAS [13]
	Cell shape	Rod	TAS [6,23]
	Motility	Non-motile	NAS [13]
	Sporulation	Nonsporulating	TAS [13]
	Temperature range	Not reported	
	Optimum temperature	70 °C	TAS [13]
	Carbon source	Several mono- and disaccharides; some organic acids and amino acids	TAS [13]
	Energy source	Chemoorganotroph	TAS [6,23]
	Terminal electron acceptor	O_2_, NO_3_^-^	TAS [6,23]
MIGS-6	Habitat	Terrestrial hot springs	TAS [6,23]
MIGS-6.3	Salinity	3.90 g/L total dissolved solids	TAS [1]
MIGS-22	Oxygen	Facultative anaerobe (nitrate reduction)	TAS [6,23]
MIGS-15	Biotic relationship	Free living	TAS [6,23]
MIGS-14	Pathogenicity	Non-pathogenic	NAS
MIGS-4	Geographic location	Sandy’s Spring West, Great Boiling Springs geothermal field, Nevada	TAS [6]
MIGS-5	Sample collection time	October, 2008	TAS [6]
MIGS-4.1 MIGS-4.2	Latitude Longitude	N40° 39.182’ W119° 22.496’	TAS [1]
MIGS-4.3	Depth	Sediment/water interface (shallow)	TAS [1]
MIGS-4.4	Altitude	1,203 m	NAS

^a^Evidence codes - IDA: Inferred from Direct Assay; TAS: Traceable Author Statement (i.e., a direct report exists in the literature); NAS: Non-traceable Author Statement (i.e., not directly observed for the living, isolated sample, but based on a generally accepted property for the species, or anecdotal evidence). These evidence codes are from Gene Ontology project [24].

**Table 1(b):** Classification and general features of *Thermus thermophilus* JL-18 according to the MIGS recommendations [13].

**MIGS ID**	**Property**	**Term**	**Evidence code**^a^
	Current classification	Domain *Bacteria*	TAS [14]
		Phylum *Deinococcus-Thermus*	TAS [15]
		Class *Deinococci*	TAS [16,17]
		Order *Thermales*	TAS [16,18]
		Family *Thermaceae*	TAS [16,19]
		Genus *Thermus*	TAS [20-22]
		Species *Thermus thermophilus*	TAS [25-27]
		Type strain JL-18	TAS [28]
	Gram stain	Negative	TAS [28]
	Cell shape	Rod	TAS [6,28]
	Motility	Non-motile	TAS [28]
	Sporulation	Nonsporulating	TAS [28]
	Temperature range	Not reported	
	Optimum temperature	70 °C	TAS [28]
	Carbon source	Several mono- and disaccharides; some organic acids and amino acids	TAS [28]
	Energy source	Chemoorganotroph	TAS [28]
	Terminal electron acceptor	O_2_, NO_3_^-^	TAS [6]
MIGS-6	Habitat	Terrestrial hot springs	TAS [6]
MIGS-6.3	Salinity	3.90 g/L total dissolved solids	TAS [1]
MIGS-22	Oxygen	Facultative anaerobe (nitrate reduction)	TAS [6,13]
MIGS-15	Biotic relationship	Free living	TAS [6,13]
MIGS-14	Pathogenicity	Non-pathogenic	NAS
MIGS-4	Geographic location	Sandy’s Spring West, Great Boiling Springs geothermal field, Nevada	TAS [6]
MIGS-5	Sample collection time	12/2008	TAS [6]
MIGS-4.1 MIGS-4.2	Latitude Longitude	N40° 39.182’ W119° 22.506’	TAS [1]
MIGS-4.3	Depth	Sediment/water interface (shallow)	TAS [1]
MIGS-4.4	Altitude	1,203 m	NAS

^a^Evidence codes - IDA: Inferred from Direct Assay; TAS: Traceable Author Statement (i.e., a direct report exists in the literature); NAS: Non-traceable Author Statement (i.e., not directly observed for the living, isolated sample, but based on a generally accepted property for the species, or anecdotal evidence). These evidence codes are from Gene Ontology project [24].

## Genome sequencing information

### Genome project history

*T. oshimai* JL-2 and *T. thermophilus* JL-18 were selected based on their important roles in denitrification and also for their biotechnological potential. The genome projects for both the organisms are deposited in the Genomes OnLine Database [29] and the complete sequences are deposited in GenBank. Sequencing, finishing, and annotation were performed by the DOE Joint Genome Institute (JGI). A summary of the project and information associated with MIGS version 2.0 compliance [13] are shown (*T. oshimai* JL-2; Table 2(a) and *T. thermophilus* JL-18; Table 2(b)).

**Table 2(a):** *Thermus oshimai* JL-2 genome sequencing project information

**MIGS ID**	**Property**	**Term**
MIGS-31	Finishing quality	Finished
MIGS-28	Libraries used	454 standard and PE, Illumina
MIGS-29	Sequencing platforms	Illumina GAii, 454-GS-FLX-Titanium
MIGS-31.2	Fold coverage	38.3× (454), 2,228.9× (Illumina)
MIGS-30	Assemblers	Newbler v 2.3 (pre-release)
MIGS-32	Gene calling method	Prodigal 1.4, GenePRIMP
	Genome Date of Release	
	Genbank ID	CP003249.1 (chromosome) CP003250.1 (Plasmid pTHEOS01) CP003251.1 (Plasmid pTHEOS02)
	Genbank Date of Release	November 5, 2012
	GOLD ID	Gc02356
	Project relevance	Biotechnological

**Table 2(b):** *Thermus thermophilus* JL-18 genome sequencing project information

**MIGS ID**	**Property**	**Term**
MIGS-31	Finishing quality	Finished
MIGS-28	Libraries used	454 standard and PE, Illumina
MIGS-29	Sequencing platforms	Illumina GAii, 454-GS-FLX-Titanium
MIGS-31.2	Fold coverage	38.1× (454), 300× (Illumina)
MIGS-30	Assemblers	Newbler v 2.3 (pre-release)
MIGS-32	Gene calling method	Prodigal 1.4, GenePRIMP
	Genome Date of Release	Oct 21, 2011
	Genbank ID	CP003252.1 (chromosome) CP003253.1 (plasmid pTTJL1801) CP003254.1 (plasmid pTTJL1802)
	Genbank Date of Release	April 9, 2012
	GOLD ID	Gc02194
	Project relevance	Biotechnological

### Growth conditions and DNA isolation

Axenic cultures of *T. oshimai* JL-2 and *T. thermophilus* JL-18 were grown aerobically on *Thermus* medium as described [6] and DNA was isolated from 0.5-1.0 g of cells using the Joint Genome Institute's (JGI) cetyltrimethyl ammonium bromide protocol [30].

### Genome sequencing and assembly

The draft genomes of *Thermus oshimai* JL-2 and *Thermus thermophilus* JL-18 were generated at the DOE Joint Genome Institute (JGI) using a combination of Illumina [31] and 454 technologies [32].

For *T. oshimai* JL-2, we constructed and sequenced an Illumina GAii shotgun library which generated 146,341,736 reads totaling 11,122 Mb, a 454 Titanium standard library which generated 181,476 reads and 1 paired end 454 library with an average insert size of 8 kb that generated 285,154 reads totaling 146.6 Mb of 454 data. For *T. thermophilus* JL-18, we constructed and sequenced an Illumina GAii shotgun library that generated 74,093,820 reads totaling 5,631.1 Mb, a 454 Titanium standard library that generated 212,217 reads and 1 paired end 454 library with an average insert size of 7 kb that generated 121,082 reads totaling 116.9 Mb of 454 data. All general aspects of library construction and sequencing performed at the JGI can be found at [30]. The initial draft assemblies of *T. oshimai* JL-2 and *T. thermophilus* JL-18 contained 39 contigs in 2 scaffolds and 75 contigs in 3 scaffolds, respectively.

The 454 Titanium standard data and the 454 paired end data were assembled together with Newbler, version 2.3-PreRelease-6/30/2009. The Newbler consensus sequences were computationally shredded into 2 kb overlapping fake reads (shreds). Illumina sequencing data was assembled with VELVET, version 1.0.13 [33], and the consensus sequence were computationally shredded into 1.5 kb overlapping fake reads (shreds). We integrated the 454 Newbler consensus shreds, the Illumina VELVET consensus shreds and the read pairs in the 454 paired end library using parallel phrap, version SPS - 4.24 (High Performance Software, LLC). The software Consed [34] was used in the following finishing process. Illumina data was used to correct potential base errors and increase consensus quality using the software Polisher developed at JGI (Alla Lapidus, unpublished). Possible mis-assemblies were corrected using gapResolution (Cliff Han, unpublished), Dupfinisher [35] or sequencing cloned bridging PCR fragments with subcloning. Gaps between contigs were closed by editing in Consed, by PCR and by Bubble PCR (J-F Cheng, unpublished) primer walks. Additional reactions were necessary to close gaps and to raise the quality of the finished sequence (*T. oshimai* JL-2: 20 reactions; *T. thermophilus* JL-18: 45).

The total size of the genomes are 2,401,329 bp (*T. oshimai* JL-2) and 2,311,212 bp (*T. thermophilus* JL-18). The final assembly of *T. oshimai* JL-2 genome is based on 91.8 Mb of 454 draft data which provides an average 38.3× coverage of the genome and 5,349.4 Mb of Illumina draft data which provides an average 2,228.9× coverage of the genome. The final assembly of *T. thermophilus* JL-18 genome is based on 87.7 Mb of 454 draft data which provides an average 38.1× coverage of the genome and 690 Mb of Illumina draft data which provides an average 300× coverage of the genome. The data and metadata are made available at the JGI Integrated Microbial Resource website (IMG) [31].

### Genome annotation

Initial identification of genes was done using Prodigal [36], a part of the DOE-JGI Annotation pipeline, followed by manual curation using GenePRIMP [37]. The predicted ORFs were translated into putative protein sequences and searched against databases including: NCBI nr, Uniprot, TIGR-Fam, Pfam, PRIAM, KEGG, COG, and Interpro. Additional annotations and curations were performed using the Integrated Microbial Genomes - Expert Review (IMG-ER) platform [33].

## Genome properties

**Figure 2:**
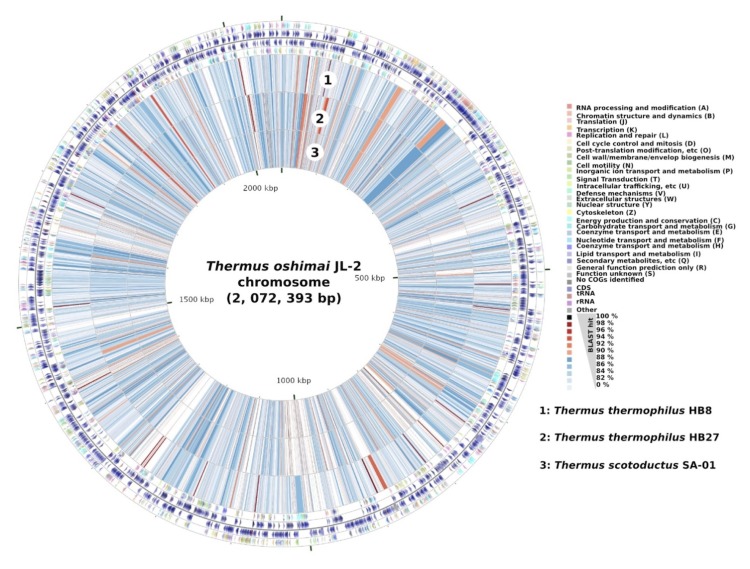
Map of *T. oshimai* JL-2 chromosome compared with other *Thermus* chromosomes. The outer four circles show the genes in forward and reverse strands and their corresponding COG categories. BLASTN hits (percentage identities) from *T. thermophilus* HB8 (1), *T. thermophilus* HB27 (2), and *T. scotoductus* SA-01 (3) chromosomes are shown in the inner three circles. Maps were created using CGView Comparison Tool [32].

The *T. oshimai* JL-2 genome includes one circular chromosome of 2,072,393 bp (2205 predicted genes), a circular megaplasmid, pTHEOS01 (0.27 Mb, 268 predicted genes), and a smaller circular plasmid, pTHEOS02 (57.2 Kb, 75 predicted genes), for a total size of 2,401,329 bp. Of the total 2,548 predicted genes, 2,488 were protein-coding genes. A total of 2,015 (79%) protein-coding genes were assigned to a putative function with the remaining annotated as hypothetical proteins. The properties and the statistics of the genome are summarized in Table 3a, Table 3b, Table 3c and Figure 2).

**Table 3(a):** Summary of *Thermus oshimai* JL-2 genome: one chromosome and two plasmids

**Label**	**Size (Mb)**	**Topology**	**INSDC identifier**	**RefSeq ID**
Chromosome	2.072393	Circular	CP003249.1	-
Plasmid pTHEOS01	0.271713	Circular	CP003250.1	-
Plasmid pTHEOS02	0.057223	Circular	CP003251.1	-

**Table 3(b):** Nucleotide content and gene count levels of *Thermus oshimai* JL-2 genome

**Attribute**	**Value**	**% of Total^a^**
Genome size (bp)	2,401,329	100.00
DNA coding region (bp)	2,251,025	93.74
DNA G+C content (bp)	1,646,250	68.56
Total genes^b^	2,548	100.00
RNA genes	60	2.35
Protein-coding genes	2,488	97.65
Pseudogenes	53	2.08
Genes in paralog clusters	1,099	43.13
Genes with function prediction	2,014	79.04
Genes assigned to COGs	2,003	78.61
Genes assigned Pfam domains	1,998	78.41
Genes with signal peptides	862	33.83
Genes with transmembrane helices	511	20.05
CRISPR repeats	5	

^a^The total is based on either the size of the genome in base pairs or the total number of protein coding genes in the annotated genome.

^b^Pseudogenes may also be counted as protein coding or RNA genes, so is not additive under total gene count.

**Table 3(c):** Number of *Thermus oshimai* JL-2 genes associated with the 25 general COG functional categories

**Code**	**Value**	**%age**^a^	**Description**
J	146	6.67	Translation
A	4	0.18	RNA processing and modification
K	114	5.21	Transcription
L	117	5.35	Replication, recombination and repair
B	2	0.09	Chromatin structure and dynamics
D	35	1.60	Cell cycle control, mitosis and meiosis
Y	0	0	Nuclear structure
V	25	1.14	Defense mechanisms
T	76	3.47	Signal transduction mechanisms
M	90	4.11	Cell wall/membrane biogenesis
N	23	1.05	Cell motility
Z	1	0.05	Cytoskeleton
W	0	0	Extracellular structures
U	44	2.01	Intracellular trafficking and secretion
O	85	3.88	Posttranslational modification, protein turnover, chaperones
C	154	7.04	Energy production and conversion
G	132	6.03	Carbohydrate transport and metabolism
E	219	10.01	Amino acid transport and metabolism
F	74	3.38	Nucleotide transport and metabolism
H	126	5.76	Coenzyme transport and metabolism
I	89	4.07	Lipid transport and metabolism
P	99	4.52	Inorganic ion transport and metabolism
Q	51	2.33	Secondary metabolites biosynthesis, transport and catabolism
R	289	13.21	General function prediction only
S	193	8.82	Function unknown
-	545	21.39	Not in COGs

^a^The total is based on the total number of protein coding genes in the annotated genome.

**Figure 3:**
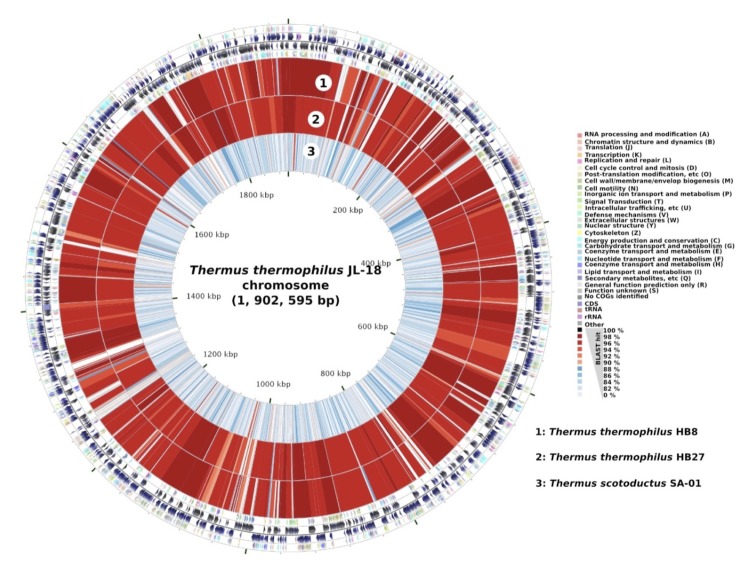
Map of *T. thermophilus* JL-18 chromosome compared with other *Thermus* chromosomes. The outer four circles show the genes in forward and reverse strands and their corresponding COG categories. BLASTN hits (percentage identities) from *T. thermophilus* HB8 (1), *T. thermophilus* HB27 (2), and *T. scotoductus* SA-01 (3) chromosomes are shown in the inner three circles. Maps were created using CGView Comparison Tool [32].

The *T. thermophilus* JL-18 genome includes one circular chromosome of 1,902,595 bp (2,057 predicted genes), a circular megaplsmid, pTTJL1801 (0.26 Mb, 279 predicted genes), and a smaller circular plasmid, pTTJL1802 (0.14 Mb, 172 predicted genes), for a total size of 2,311,212 bp. Of the total 2,508 predicted genes, 2,452 were protein-coding genes. A total of 1,979 (79%) of protein-coding genes were assigned to a putative function with the remaining annotated as hypothetical proteins. The properties and the statistics of the genome are summarized in Table 4a, Table 4b, Table 4c and Figure 3.

**Table 4a:** Summary of *Thermus thermophilus* JL-18 genome: one chromosome and two plasmids

**Label**	**Size (Mb)**	**Topology**	**INSDC identifier**	**RefSeq ID**
Chromosome	1.902595	Circular	CP003252.1	NC_017587.1
Plasmid pTTJL1801	0.265886	Circular	CP003253.1	NC_017588.1
Plasmid pTTJL1802	0.0142731	Circular	CP003254.1	NC_017590.1

**Table 4b:** Nucleotide content and gene count levels of *Thermus thermophilus* JL-18 genome

**Attribute**	**Value**	**% of total^a^**
Genome size (bp)	2,311,212	100.00
DNA coding region (bp)	2,172,588	94.00
DNA G+C content (bp)	1,594,227	68.98
Total genes^b^	2,508	100.00
RNA genes	56	2.23
Protein-coding genes	2,452	97.77
Pseudogenes	50	1.99
Genes in paralog clusters	1,069	42.62
Genes with function prediction	1,979	78.91
Genes assigned to COGs	1,992	79.43
Genes assigned Pfam domains	1,962	78.23
Genes with signal peptides	464	18.5
Genes with transmembrane helices	518	20.65
CRISPR repeats	3	

^a^The total is based on either the size of the genome in base pairs or the total number of protein coding genes in the annotated genome.

^b^Pseudogenes may also be counted as protein coding or RNA genes, so is not additive under total gene count.

**Table 4c:** Number of *Thermus thermophilus* JL-18 genes associated with the 25 general COG functional categories

**Code**	**Value**	**%age**^a^	**Description**
J	148	6.79	Translation
A	1	0.05	RNA processing and modification
K	104	4.77	Transcription
L	130	5.97	Replication, recombination and repair
B	2	0.09	Chromatin structure and dynamics
D	33	1.51	Cell cycle control, mitosis and meiosis
Y	0	0	Nuclear structure
V	25	1.15	Defense mechanisms
T	67	3.07	Signal transduction mechanisms
M	87	3.99	Cell wall/membrane biogenesis
N	30	1.38	Cell motility
Z	1	0.05	Cytoskeleton
W	0	0	Extracellular structures
U	57	2.62	Intracellular trafficking and secretion
O	82	3.76	Posttranslational modification, protein turnover, chaperones
C	149	6.84	Energy production and conversion
G	125	5.74	Carbohydrate transport and metabolism
E	216	9.91	Amino acid transport and metabolism
F	64	2.94	Nucleotide transport and metabolism
H	119	5.46	Coenzyme transport and metabolism
I	94	4.31	Lipid transport and metabolism
P	96	4.41	Inorganic ion transport and metabolism
Q	57	2.62	Secondary metabolites biosynthesis, transport and catabolism
R	291	13.35	General function prediction only
S	201	9.22	Function unknown
-	516	20.57	Not in COGs

^a^The total is based on the total number of protein coding genes in the annotated genome.

## Comparison with other sequenced genomes

The chromosome of *T. thermophilus* JL-18 was compared with the chromosomes of *T. thermophilus* strains HB8 and HB27 [38] using nucmer [39]. The megaplasmid pTTJL1801 was also compared with the megaplasmid sequences of HB8 and HB27. Dot plot results from this analysis (Figure 4(a)) demonstrate a high degree of synteny between the chromosomes of JL-18, HB8, and HB27; however, little synteny exists between the megaplasmids. *T. oshimai* JL-2 chromosome and megaplasmid sequences were also compared with those of *T. thermophilus* JL-18; however, little very synteny was apparent (Figure 4(b)).

**Figure 4(a):**
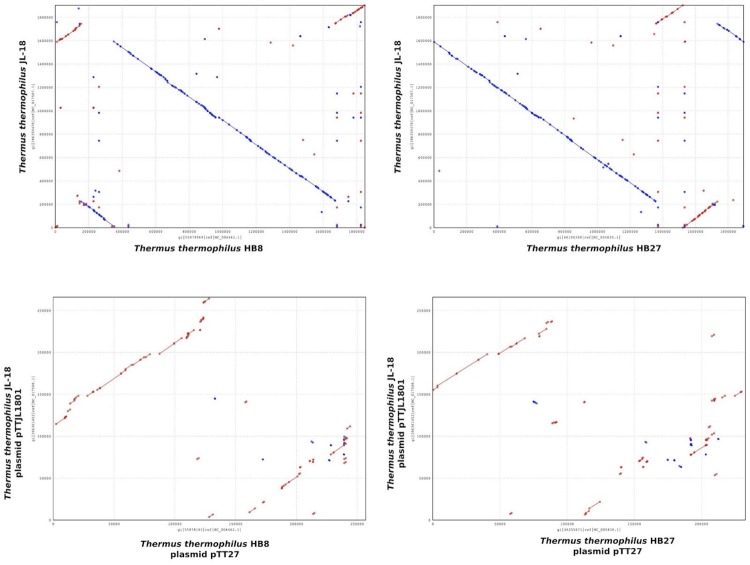
Dot plot comparison of *T. thermophilus* JL-18 chromosome and megaplasmid DNA sequence with those of the strains HB8 and HB27.

**Figure 4(b):**
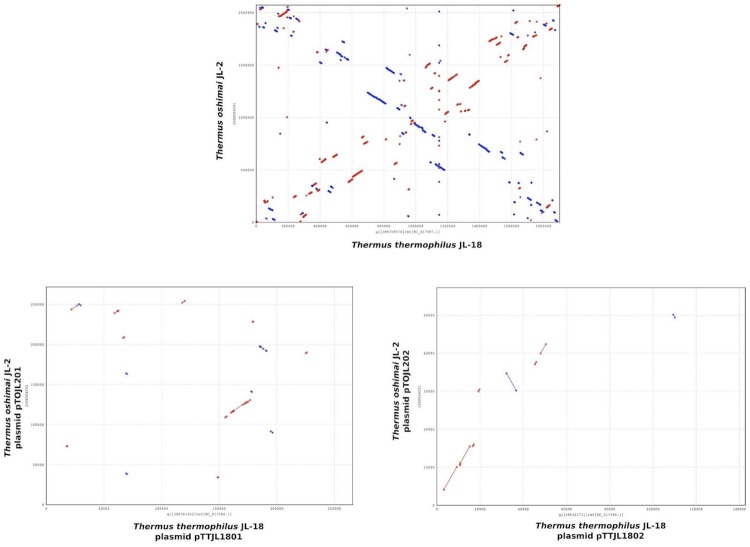
Dot plot comparing the chromosome and megaplasmid DNA sequence of *T. oshimai* JL-2 and *T. thermophilus* JL-18.

## Profiles of metabolic networks and pathways

**Figure 5:**
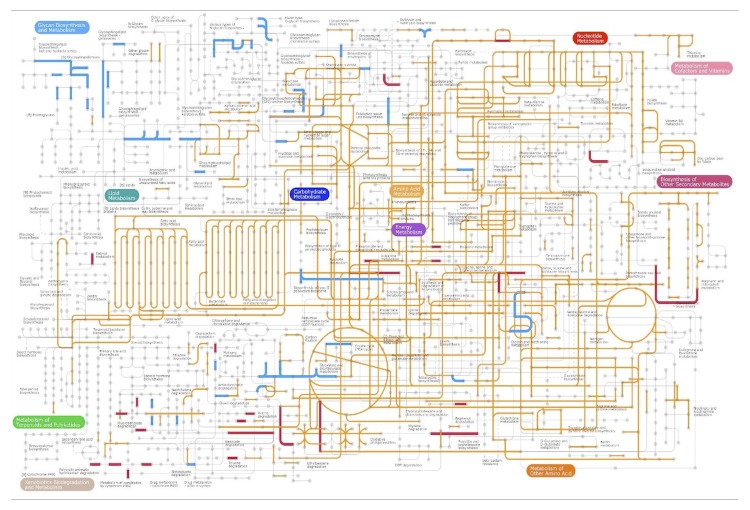
Metabolic pathways identified using iPATH2 [40]. Orange lines are common pathways that were identified in *T. oshimai* JL-2 and *T. thermophilus* JL-18. Blue lines indicate pathways unique to *T. oshimai* JL-2 and red lines indicate pathways unique to *T. thermophilus* JL-18.

*T. oshimai* JL-2 and *T. thermophilus* JL-18 genomes encode genes for complete glycolysis, tricarboxylic acid (TCA) cycle, and pentose phosphate pathway (Figure 5). The genomes also encode glucosidases, glycosidases, proteases, and peptidases, highlighting the ability of these species to use various carbohydrate and peptide substrates. Thus, central carbon metabolic pathways are very similar to those of *T. thermophilus* HB27 [38] and *T. scotoductus* SA-01 [41].

## Genes involved in denitrification

Denitrification involves the conversion of nitrate to dinitrogen through the intermediates nitrite, nitric oxide, and nitrous oxide and is mediated by *nar*, *nir*, *nor*, and *nos* genes [4]. Incomplete denitrification phenotypes terminating in the production of nitrous oxide have recently been reported for a large number of *Thermus* isolates, including *T. oshimai* JL-2 and *T. thermophilus* JL-18 [6].

**Figure 6:**
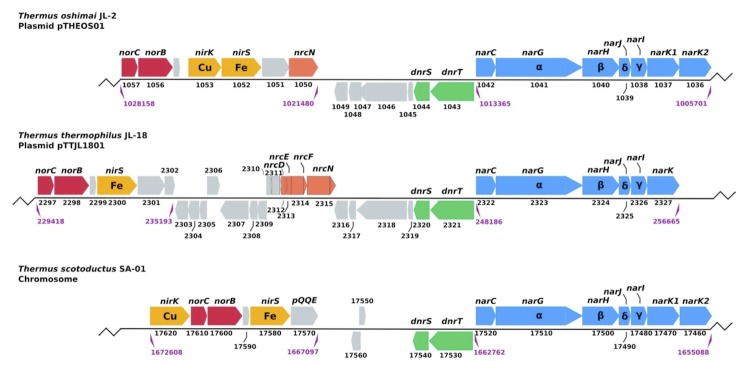
Map showing the organization of *nar* operon and neighboring genes involved in denitrification located on the megaplasmids of *T. oshimai* JL-2 (pTHEOS01) and *T. thermophilus*JL-18 (pTTJL1801) and the chromosome of *T. scotoductus* SA-01. Fe: heme protein-containing nitrite reductase, Cu: copper-containing nitrite reductase. Numbers below the genes indicate the provisional ORF numbers in *T. oshimai* JL-2 (Theos_1057 - Theos_1036) and *T. thermophilus* JL-18 (TtJL18_2297 to TtJL18_2327), the locations in the megaplasmid are indicated below. *nar*: nitrate reductase; *nir*: nitrite reductase; *nos*: nitric oxidereductase; *dnr*: denitrification regulator [41-43].

Figure 6 shows the organization of the *nar* operon and neighboring genes involved in denitrification in *T. oshimai* JL-2, *T. thermophilus* JL-18, and *T. scotoductus* SA-01. These gene clusters are located on the megaplasmids of *T. oshimai* JL-2 and *T. thermophilus* JL-18, as in other *T. thermophilus* strains [44,45]. They are located on the chromosome in *T. scotoductus* SA-01 [41]. The *nar* operons show a high degree of synteny and all include genes encoding the membrane-bound nitrate reductase (NarGHI), the associated periplasmic cytochrome NarC, and the dedicated chaperone NarJ. All three strains contained homologs of NarK1, which is a member of the major facilitator superfamily that likely functions as a nitrate/proton symporter [46,47]. However, some experiments in *T. thermophilus* HB8 suggest NarK1 might also function in nitrite extrusion [39]. *T. oshimai* JL-2 and *T. scotoductus* SA-01 also contain homologs of NarK2 (annotated as *nep* in *T. scotoductus* SA-01 [41]), which likely encodes a nitrate/nitrite antiporter [44,48]. No significant BLASTP hits for periplasmic nitrate reductase subunits NapB and NapC were found in *T. oshimai* JL-2 and *T. thermophilus* JL-18, consistent with the use of the Nar system in the *Thermales*.

All three strains contain a *dnrST* operon adjacent to, but divergently transcribed from, the *narGHJIK* operon. *dnrST* encodes transcriptional activators responsible for upregulation of the nitrate respiration pathway in the absence of O_2_ and the presence of nitrogen oxides or oxyanions [42] (Figure 6).

Both the species contain a putative *nirK,* which encodes the NO-forming, Cu-containing nitrite reductase. In addition, *T. oshimai* JL-2 and *T. scotoductus* SA-01 both harbor *nirS* [41], which encodes the isofunctional tetraheme cytochrome *cd*_1_-containing nitrite reductase. Previous studies have suggested that bacteria use either NirK or NirS, but not both, for the reduction of nitrite [49]. The unique presence of NirK and NirS in *T. oshimai* JL-2 and *T. scotoductus* SA-01 likely enhances their denitrification abilities since isoenzymes are typically kinetically distinct and/or regulated differently. This idea is consistent with the distinct denitrification phenotypes of *T. oshimai* strains as compared to *T. thermophilus* strains reported previously, including strains *T. oshimai* JL-2 and *T. thermophilus* JL-18 [6]. In those studies, nitrite accumulated in the medium at concentrations of <150 µM in *T. thermophilus* strains, whereas it was rapidly produced to concentrations >200 µM but consumed rapidly to below method detection limits in *T. oshimai* strains.

**Figure 7:**
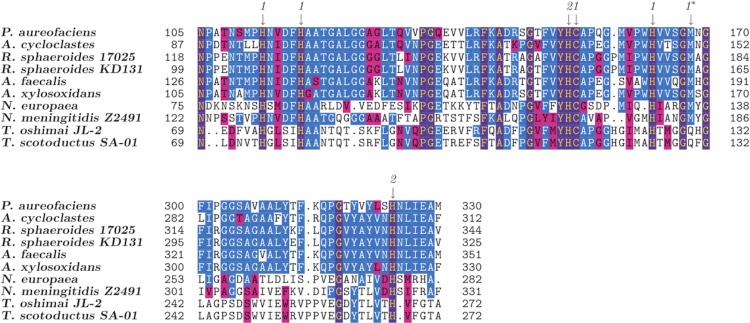
***Thermus oshimai* JL-2 gene Theos_1053 encodes a Copper-containing nitrite reductase**. Amino acid sequences of known Cu-containing nitrite reductases from *Pseudomonas aureofaciens* (*P. aureofaciens,* GI: 287907), *Achromobacter cycloclastes* (*A. cycloclastes*, GI: 157835402), *Rhodobacter sphaeroides* ATCC 17025 (*R. sphaeroides* 17025, GI: 146277634), *Rhodobacter sphaeroides* KD131 (*R. sphaeroides* KD131, GI: 221638756), *Alcaligenes faecalis* (*A. faecalis*, GI: 393758960), *Alcaligenes xylosoxidans* (*A. xylosoxidans*, GI: 422318032), *Nitrosomonas europaea* (*N. europaea*, GI: 30248928), *Neisseria meningitidis* Z2491 (*N. meningitidis* Z2491, GI: 218768658) and *Thermus scotoductus* SA-01 (*T. scotoductus* SA-01, GI: 320450829) were aligned using Muscle v3.8.31 [51] along with *Thermus oshimai* JL-2 (*T. oshimai* JL-2, GI: 410732282) Theos_1053. Putative copper-binding residues are indicated with downward arrows according to their classes: 1: type 1 (blue) Cu; 2: type 2 (nonblue) Cu [49]. Numbers on left and right of the alignments refer to positions in the alignment. Asterisk (*) indicates the M→Q substitution in *T. oshimai* JL-2 and *T. scotoductus* SA-01.

NirK functions as a homo-trimer [50] and contains type 1 (blue) and type 2 (non-blue) copper-binding residues [49]. Comparison of the NirK from *T. oshimai* JL-2 and *T. scotoductus* SA-01 with previously studied NirK amino acid sequences revealed that six of the seven copper-binding residues are conserved, except for a single methionine (M) to glutamine (Q) substitution in both *Thermus* proteins (Figure 7; indicated by an asterisk (*)). Glutamine, not methionine, is the copper-binding ligand in the case of stellacyanin, a blue (type 1) copper-containing protein [52,53]. A M121Q recombinant protein of *Alcaligenes denitrificans* azurin showed similar electron paramagnetic resonance (EPR), but exhibited a 100-fold lower redox activity when compared to wild-type azurin [54]. Therefore, although the methionine is replaced with a glutamine in the *T. oshimai* JL-2 NirK, it is possible that this glutamine residue can function as a copper-binding ligand similar to stellacyanin and azurin. The large and small subunits of nitric oxide reductase (NorB and NorC) are predicted to be co-transcribed along with nitrite reductases in *T. oshimai* JL-2, *T. thermophilus* JL-18 and *T. scotoductus* SA-01 (Figure 6).

Genes encoding the 15 subunit NADH-quinone oxidoreductase [55] were identified in both genomes (Theos_0703 to 0716, 1811 in *T. oshimai* JL-2; TTJL18_1786 to 1799, 1580 *T. thermophilus* JL-18). *nrcDEFN*, a four gene operon encoding a novel NADH dehydrogenase, is adjacent to the *nar* operon in the megaplasmid of *T. thermophilus* HB8 and has been previously implicated in nitrate reduction [43]. In *T. thermophilus* JL-18, the operon is present (Figure 6), although (TTJL18_2313) is truncated (NarE in HB8: 232 AA, in JL-18: 78 AA). In *T. oshimai* JL-2, only *nrcN* is present. Theos_0161 and Theos_0162, orthologs of *Wolinella succinogenes* NrfA and NrfH [56], respectively, were identified in *T. oshimai* JL-2 suggesting that *T. oshimai* JL-2 may be capable of respiratory nitrite ammonification, although this phenotype has not yet been observed in *Thermus* [6].

Other possible electron transport components include a *ba*_3_-type heme-copper oxidase (Theos_1499, 1498, 1497, *T. oshimai* JL-2; TTJL18_0925, 0926, 0927 *T. thermophilus* JL-18) and *bc*_1_ complex encoded by the *FbcCDFB* operon [57]. (Theos_0106 to 0109, *T. oshimai* JL-2; TTJL18_2018 to 2021 *T. thermophilus* JL-18). In addition, both *T. oshimai* JL-2 and *T. thermophilus* JL-18 harbor genes for archaeal-type V_0_-V_1_ (vacuolar) type ATPases, which appears to have been acquired from *Archaea* prior to the divergence of the modern *Thermales* [58].

### Genes involved in iron reduction

*T. scotoductus* SA-01 has been reported to be capable of dissimilatory Fe^3+^ reduction; however, the biochemical basis of iron reduction has not been elucidated in *Thermus* [41,59]. Sequences of proteins involved in iron reduction [60] in *Shewanella oneidensis* MR-1 (MtrA, MtrF, OmcA) and *Geobacter sulfurreducens* KN400 (OmcB, OmcE, OmcS, OmcT, OmcZ) were used as search queries into *Thermus* genomes using BLASTP. No hits were found in *T. oshimai* JL-2, *T. thermophilus* JL-18, or *T. scotoductus* SA-01. This suggests that the biochemical basis of iron reduction is distinct in *Thermus* compared to *Shewanella* and *Geobacter,* and offers no predictive information on whether *T. oshimai* JL-2 and *T. thermophilus* JL-18 may be able to respire iron.

### Genes involved in sulfur oxidation

A complete *sox* cluster comprising of 15 genes, including *soxCD*, is present in *T. oshimai* JL-2 and *T. thermophilus* JL-18 genomes. SoxCD is essential for chemotrophic growth of *P. pantotrophus* [61]. Taken together, this suggests that *T. oshimai* JL-2 and *T. thermophilus* JL-18 may use thiosulfate as an electron donor and are similar to other sulfur-oxidizing *Thermus* strains including *T. scotoductus* IT-7254 [62] and *T. scotoductus* SA-01 [41]. Other *T. thermophilus* genomes also harbor this gene cluster, suggesting thiosulfate oxidation may be widely distributed in *Thermus* [38].

A variety of chemotrophs and anoxygenic phototrophs can oxidize hydrogen sulfide, organic sulfur compounds, sulfite, and thiosulfate as electron donors for respiration [63]. Reconstituted proteins of SoxXA, SoxYZ, SoxB and SoxCD together, but not alone, mediate the oxidation of thiosulfate, sulfite, sulfur, and hydrogen sulfide in *Paratrophus pantotrophus* [61]. The absence of free intermediates of sulfur oxidation and the occurrence of sulfite oxidation without SoxCD in *P. pantotrophus* excludes SoxCD as a sulfite dehydrogenase and provides evidence to its role as a sulfur dehydrogenase with protein-bound sulfur atom [61].

### Polysulfide reductase in *T. oshimai* JL-2

In *T. oshimai* JL-2, three proteins showed high sequence identity to PsrA (88%; Theos_0751), PsrB (86%; Theos_0750), and PsrC (83%; Theos_0749) of *T. thermophilus* HB27, which is likely involved in anaerobic respiration using polysulfide as a terminal electron acceptor. In *T. thermophilus* HB27, PsrA is the putative catalytic subunit containing two molybdopterin guanine dinucleotide co-factors and a cubane-type [4Fe-4S] cluster. Electron transfer is likely mediated by PsrB, which also contains a [4Fe-4S] cluster, while PsrC is a putative transmembrane protein that contains the electron carrier menaquinone-7 (MK-7). PSR functions as a hexamer (composed of 2 subunits each of A, B and C) and catalyzes the reactions: MKH_2_→MK + 2H^+^ + 2e^-^ in the membrane, and S_n_^2-^+ 2e^-^ + 2H^+^ + S_n-1_^2-^ + H_2_S in the periplasm [64]. However, the *Thermus* PsrABC proteins exhibit very low identity to *Wolinella succinogenes* PsrABC proteins that have been functionally characterized (PsrA: 33%, PsrB 46%, no clear BLASTP hits found in *T. oshimai* JL-2 for *W. succinogenes* PsrC) [65]. In *Wolinella succinogenes*, formate dehydrogenase or hydrogenase and polysulfide reductase form the electron transport chain and mediate the reduction of polysulfide with formate or H_2_ [64]. In *T. oshimai* JL-2, Theos_1377 encodes a putative formate dehydrogenase alpha subunit. Another gene, Theos_1111, encodes a putative formate dehydrogenase family accessory protein (FdhD), which is required for regulation of the formate dehydrogenase catalytic subunit [66] and is conserved in many members of the *Thermaceae*, including *T. scotoductus* SA-01 (TSC_c10040). Although the genes needed for polysulfide reduction are present, polysulfide reduction in *T. oshimai* JL-2 has not been tested.

### Genes involved in DNA uptake

A significant number of genes in hyperthermophilic bacteria are of archaeal origin, and appear to have been acquired through inter-domain gene transfer [67], which is mediated by both transformation and conjugation systems [68]. *T. thermophilus* HB27 is naturally competent to both linear and circular DNA, and DNA transport mechanisms in this species have been well studied [69,70]. The genome of *T. oshimai* JL-2 and *T. thermophilus* JL-18 both contain homologs of DNA transport genes (Table 5), suggesting that both *T. oshimai* JL-2 and *T. thermophilus* JL-18 are naturally competent.

**Table 5:** Identification of competence proteins in *T. oshimai* JL-2 and *T. thermophilus* JL-18 by IMG/ER [71].^†^

**Known competence** **proteins in HB27**	***T. oshimai* JL-2**	***T. thermophilus* JL-18**	**Potential Function**
ComEC	Theos_2202	TtJL18_2054	DNA transport through the IM
ComEA	Theos_2201	TtJL18_2053	DNA binding
DprA	Theos_0224	TtJL18_1834	Transport of ssDNA to RecA
PilA1	Theos_1235, Theos_1236	TtJL18_0836, TtJL18_0835	Structural subunits
PilA2	Theos_1237	TtJL18_0834	Structural subunits
PilA3	Theos_1238	TtJL18_0833	Structural subunits
PilA4	Theos_1240	TtJL18_0837	Structural subunits
PilD	Theos_1920	TtJL18_0122	Export and maturation of prepilins
PilF	Theos_1970	TtJL18_0018	Retraction of pili proteins and DNA translocation
PilC	Theos_0570	TtJL18_1257	Linkage of periplasmic and cytoplasmic proteins
PilQ	Theos_0435	TtJL18_0665	Directing DNA transporter through OM
ComZ	Theos_1239	TtJL18_0832	IM protein, function unknown
PilM	Theos_0439	TtJL18_0669	ATPase, function unknown
PilN	Theos_0438	TtJL18_0668	IM protein, function unknown
PilO	Theos_0437	TtJL18_0667	IM protein, function unknown
PilW	Theos_0436	TtJL18_0666	OM protein, stabilization of PilQ

^†^BLASTP analysis using sequences of known competence proteins from *T. thermophilus* HB27 as queries. Table modified from [72].

## Conclusions

We report the finished genomes of *T. oshimai* JL-2 and *T. thermophilus* JL-18. *T. oshimai* JL-2 is the first complete genome to be reported for this species, while *T. thermophilus* JL-18 is the fourth genome to be reported for *T. thermophilus*. Analysis of the genomes revealed that they encode enzymes for the reduction of nitrate to nitrous oxide, which is consistent with the high flux of nitrous oxide reported in GBS [6], and explains the truncated denitrification phenotype reported for many *Thermus* isolates obtained from that system [6]. It is intriguing that *Thermus scotoductus* SA-01 also has genes encoding the sequential reduction of nitrate to nitrous oxide but lacks genes encoding the nitrous oxide reductase. The high degree of synteny in the respiratory gene cluster combined with the conserved absence of the nitrous oxide reductase suggests incomplete denitrification might be a previously unrecognized but conserved feature of denitrification pathways in the genus *Thermus*, although *T. thermophilus* NAR1 appears to be capable of complete denitrification to N_2_ [73]. Another unusual feature of the *T. oshimai* JL-2 and *T. scotoductus* SA-01 denitrification systems is the apparent presence of the NO-forming, Cu-containing nitrite reductase, NirK, and the isofunctional tetraheme cytochrome *cd*_1_-containing nitrite reductase, NirS.

*T. oshimai* JL-2 and *T. thermophilus* JL-18 also may be capable of sulfur oxidation since they both encode a complete, chromosomal *sox* cluster. However, experiments with GBS sediments failed to demonstrate a stimulation of denitrification when thiosulfate was added in excess [74], suggesting thiosulfate oxidation may not be coupled to denitrification in these organisms. The presence of *psrA*, *psrB* and *psrC* genes encoding polysulfide reducatase in *T. oshimai* JL-2 suggests the ability to reduce polysulfide. The function of these putative pathways could be tested with pure cultures in the laboratory.

The presence of complete macromolecular machinery for natural competence and the presence of megaplasmids harboring genes for nitrate/nitrite reduction and thermophily points out that *T. oshimai* JL-2 and *T. thermophilus* JL-18 could have acquired innumerable genes through intra- and inter-domain gene transfer, and suggests considerable plasticity in denitrification pathways. Considering the importance of these organisms in the nitrogen biogeochemical cycle, and their potential as sources of enzymes for biotechnology applications, the complete genome sequences of *T. oshimai* JL-2 and *T. thermophilus* JL-18 are valuable resources for both basic and applied research.

## References

[1] Costa KC Navarro JB Shock EL Zhang CL Soukup D Hedlund BP Microbiology and geochemistry of great boiling and mud hot springs in the United States Great Basin. Extremophiles 2009; 13:447-459 10.1007/s00792-009-0230-x 19247786

[2] Huang Z Hedlund BP Wiegel J Zhou J Zhang CL Molecular phylogeny of uncultivated Crenarchaeota in Great Basin hot springs of moderately elevated temperature. Geomicrobiol J 2007; 24:535-542 10.1080/01490450701572523

[3] Miller-Coleman RL Dodsworth JA Ross CA Shock EL Williams AJ Hartnett HE McDonald AI Havig JR Hedlund BP Korarchaeota diversity, biogeography, and abundance in Yellowstone and Great Basin hot springs and ecological niche modeling based on machine learning. PLoS ONE 2012; 7:e35964 10.1371/journal.pone.0035964 22574130 PMC3344838

[4] Zhang CL Ye Q Huang Z Li WJ Chen J Song Z Zhao W Bagwell C Inskeep WP Gao L Global occurrence and biogeography of putative archaeal *amoA* genes in terrestrial hot springs. Appl Environ Microbiol 2008; 74:6417-6426 10.1128/AEM.00843-08 18676703 PMC2570307

[5] Dodsworth JA Hungate BA Hedlund BP Ammonia oxidation, denitrification and dissimilatory nitrate reduction to ammonium in two US Great Basin hot springs with abundant ammonia-oxidizing archaea. Environ Microbiol 2011; 13:2371-2386 10.1111/j.1462-2920.2011.02508.x 21631688

[6] Hedlund BP McDonald AI Lam J Dodsworth JA Brown JR Hungate BA Potential role of *Thermus thermophilus* and *T. oshimai* in high rates of nitrous oxide (N_2_O) production in ~80 °C hot springs in the US Great Basin. Geobiology 2011; 9:471-480 10.1111/j.1472-4669.2011.00295.x 21951553

[7] Lefèvre CT Abreu F Schmidt ML Lins U Frankel RB Hedlund BP Bazylinski DA Moderately thermophilic magnetotactic bacteria from hot springs in Nevada USA. Appl Environ Microbiol 2010; 76:3740-3743 10.1128/AEM.03018-09 20382815 PMC2876459

[8] Dodsworth JA Hungate B de la Torre JR Jiang H Hedlund BP Measuring nitrification, denitrification, and related biomarkers in continental geothermal ecosystems. Methods Enzymol 2011; 486:171-203 10.1016/B978-0-12-381294-0.00008-0 21185436

[9] Cole JK Peacock JP Dodsworth JA Williams AJ Thompson DB Dong H Wu G Hedlund BP Sediment Microbial Communities in Great Boiling Spring are Controlled by Temperature and Distinct from Water Communities. [In press]. ISME J 2013 10.1038/ismej.2012.157 PMC3605714 23235293

[10] Wu M Eisen JA A simple, fast and accurate method of phylogenomic inference. Genome Biol 2008; 9:R151 10.1186/gb-2008-9-10-r151 18851752 PMC2760878

[11] Stamatakis A RAxML-VI-HPC: maximum likelihood-based phylogenetic analyses with thousands of taxa and mixed models. Bioinformatics 2006; 22:2688-2690 10.1093/bioinformatics/btl446 16928733

[12] Letunic I Bork P Interactive Tree Of Life (iTOL): an online tool for phylogenetic tree display and annotation. Bioinformatics 2007; 23:127-128 10.1093/bioinformatics/btl529 17050570

[13] Field D Garrity G Gray T Morrison N Selengut J Sterk P Tatusova T Thomson N Allen MJ Angiuoli SV The minimum information about a genome sequence (MIGS) specification. Nat Biotechnol 2008; 26:541-547 10.1038/nbt1360 18464787 PMC2409278

[14] Woese CR Kandler O Wheelis ML Towards a natural system of organisms: proposal for the domains Archaea, Bacteria, and Eucarya. Proc Natl Acad Sci USA 1990; 87:4576-4579 10.1073/pnas.87.12.4576 2112744 PMC54159

[15] Weisburg WG Giovannoni SJ Woese CR The Deinococcus-Thermus phylum and the effect of rRNA composition on phylogenetic tree construction. Syst Appl Microbiol 1989; 11:128-134 10.1016/S0723-2020(89)80051-7 11542160

[16] Validation List no. 85. Validation of publication of new names and new combinations previously effectively published outside the IJSEM. Int J Syst Evol Microbiol 2002; 52:685-690 10.1099/ijs.0.02358-0 12054225

[17] Garrity GM, Holt JG. Class I. Deinococci class. nov. In: Garrity GM, Boone DR, Castenholz RW (eds), Bergey's Manual of Systematic Bacteriology, Second Edition, Volume 1, Springer, New York, 2001, p. 395.

[18] Rainey FA, da Costa MS. Order II. Thermales ord. nov. In: Garrity GM, Boone DR, Castenholz RW (eds), Bergey's Manual of Systematic Bacteriology, Second Edition, Volume 1, Springer, New York, 2001, p. 403.

[19] da Costa MS, Rainey FA. Family I. Thermaceae fam. nov. In: Garrity GM, Boone DR, Castenholz RW (eds), Bergey's Manual of Systematic Bacteriology, Second Edition, Volume 1, Springer, New York, 2001, p. 403-404.

[20] Skerman VBD McGowan V Sneath PHA Approved Lists of Bacterial Names. Int J Syst Bacteriol 1980; 30:225-420 10.1099/00207713-30-1-225 20806452

[21] Brock TD Freeze H Thermus aquaticus gen. n. and sp. n., a nonsporulating extreme thermophile. J Bacteriol 1969; 98:289-297 5781580 10.1128/jb.98.1.289-297.1969 PMC249935

[22] Nobre MF Trüper HG da Costa MS Transfer of *Thermus ruber* (Loginova et al. 1984), *Thermus silvanus* (Tenreiro et al. 1995), and *Thermus chliarophilus* (Tenreiro et al. 1995) to *Meiothermus* gen. nov. as *Meiothermus ruber* comb. nov., *Meiothermus silvanus* comb. nov., and *Meiothermus chliarophilus* comb. nov., respectively, and emendation of the genus *Thermus.* Int J Syst Bacteriol 1996; 46:604-606 10.1099/00207713-46-2-604

[23] Williams RA Smith KE Welch SG Micallef J *Thermus oshimai* sp. nov., isolated from hot springs in Portugal, Iceland, and the Azores, and comment on the concept of a limited geographical distribution of *Thermus* species. Int J Syst Bacteriol 1996; 46:403-408 10.1099/00207713-46-2-403 8934898

[24] Ashburner M Ball CA Blake JA Botstein D Butler H Cherry JM Davis AP Dolinski K Dwight SS Eppig JT Gene ontology: tool for the unification of biology. The Gene Ontology Consortium. Nat Genet 2000; 25:25-29 10.1038/75556 10802651 PMC3037419

[25] Validation List no. 54. Validation of the publication of new names and new combinations previously effectively published outside the IJSB. Int J Syst Bacteriol 1995; 45:619-620 10.1099/00207713-45-3-619

[26] Manaia CM Hoste B Gutierrez MC Gillis M Ventosa A Kersters K da Costa MS Halotolerant Thermus strains from marine and terrestrial hot springs belong to Thermus thermophilus, ex Oshima and Imahori, 1974 nom. rev. emend. Syst Appl Microbiol 1994; 17:526-532 10.1016/S0723-2020(11)80072-X

[27] Oshima T Imahori K Description of Thermus thermophilus (Yoshida and Oshima) comb. nov. a nonsporulating thermophilic bacterium from a Japanese thermal spa. Int J Syst Bacteriol 1974; 24:102-112 10.1099/00207713-24-1-102

[28] da Costa MS, Nobre MF, Rainey FA. Genus I. *Thermus* brock and freeze 1969, 295^AL^, emend. Nobre, Trüper, and da Costa 1996b, 605, p.404-414. *In* Boone, D., Castenholz, R., and Garrity, G. (ed.), *Bergey's Manual of Systematic Bacteriology, 2nd ed. Springer-Verlag, New York, N.Y*; 2001.

[29] Pagani I Liolios K Jansson J Chen IM Smirnova T Nosrat B Markowitz VM Kyrpides NC The Genomes OnLine Database (GOLD) v.4: status of genomic and metagenomic projects and their associated metadata. Nucleic Acids Res 2012; 40:D571-D579 10.1093/nar/gkr1100 22135293 PMC3245063

[30] DOE Joint Genome Institute http://my.jgi.doe.gov/general/

[31] Bennett S Solexa Ltd. Pharmacogenomics 2004; 5:433-438 10.1517/14622416.5.4.433 15165179

[32] Ewing B Green P Base-calling of automated sequencer traces using Phred. II. Error probabilities. Genome Res 1998; 8:186-194 9521922

[33] Zerbino DR Birney E Velvet: algorithms for de novo short read assembly using de Bruijn graphs. Genome Res 2008; 18:821-829 10.1101/gr.074492.107 18349386 PMC2336801

[34] Gordon D Abajian C Green P Consed: a graphical tool for sequence finishing. Genome Res 1998; 8:195-202 9521923 10.1101/gr.8.3.195

[35] Han C, Chain P. 2006. Finishing repeat regions automatically with Dupfinisher. In Proceeding of the 2006 international conference on bioinformatics & computational biology. Hamid R. Arabnia & Homayoun Valafar (Eds), *CSREA Press* 2006:141-146.

[36] Hyatt D Chen GL Locascio PF Land ML Larimer FW Hauser LJ Prodigal: prokaryotic gene recognition and translation initiation site identification. BMC Bioinformatics 2010; 11:119 10.1186/1471-2105-11-119 20211023 PMC2848648

[37] Pati A Ivanova NN Mikhailova N Ovchinnikova G Hooper SD Lykidis A Kyrpides NC GenePRIMP: a gene prediction improvement pipeline for prokaryotic genomes. Nat Methods 2010; 7:455-457 10.1038/nmeth.1457 20436475

[38] Henne A Brüggemann H Raasch C Wiezer A Hartsch T Liesegang H Johann A Lienard T Gohl O Martinez-Arias R The genome sequence of the extreme thermophile *Thermus thermophilus.* Nat Biotechnol 2004; 22:547-553 10.1038/nbt956 15064768

[39] Kurtz S Phillippy A Delcher AL Smoot M Shumway M Antonescu C Salzberg SL Versatile and open software for comparing large genomes. Genome Biol 2004; 5:R12 10.1186/gb-2004-5-2-r12 14759262 PMC395750

[40] Yamada T Letunic I Okuda S Kanehisa M Bork P iPath2.0: interactive pathway explorer. Nucleic Acids Res 2011; 39:W412-W415 10.1093/nar/gkr313 21546551 PMC3125749

[41] Gounder K Brzuszkiewicz E Liesegang H Wollherr A Daniel R Gottschalk G Reva O Kumwenda B Srivastava M Bricio C Berenguer. Sequence of the hyperplastic genome of the naturally competent *Thermus scotoductus* SA-01. BMC Genomics 2011; 12:577 10.1186/1471-2164-12-577 22115438 PMC3235269

[42] Cava F Laptenko O Borukhov S Chahlafi Z Blas-Galindo E Gómez-Puertas P Berenguer J Control of the respiratory metabolism of *Thermus thermophilus* by the nitrate respiration conjugative element NCE. Mol Microbiol 2007; 64:630-646 10.1111/j.1365-2958.2007.05687.x 17462013

[43] Cava F Zafra O Magalon A Blasco F Berenguer J A new type of NADH dehydrogenase specific for nitrate respiration in the extreme thermophile *Thermus thermophilus.* J Biol Chem 2004; 279:45369-45378 10.1074/jbc.M404785200 15292214

[44] Ramírez-Arcos S Fernández-Herrero LA Marín I Berenguer J Two nitrate/nitrite transporters are encoded within the mobilizable plasmid for nitrate respiration of *Thermus thermophilus* HB8. J Bacteriol 2000; 182:2179-2183 10.1128/JB.182.8.2179-2183.2000 10735860 PMC111266

[45] Brüggemann H Chen C Comparative genomics of *Thermus thermophilus*: Plasticity of the megaplasmid and its contribution to a thermophilic lifestyle. J Biotechnol 2006; 124:654-661 10.1016/j.jbiotec.2006.03.043 16713647

[46] Moir JW Wood NJ Nitrate and nitrite transport in bacteria. Cell Mol Life Sci 2001; 58:215-224 10.1007/PL00000849 11289303 PMC11146482

[47] Wood NJ Alizadeh T Richardson DJ Ferguson SJ Moir JW Two domains of a dual-function NarK protein are required for nitrate uptake, the first step of denitrification in *Paracoccus pantotrophus.* Mol Microbiol 2002; 44:157-170 10.1046/j.1365-2958.2002.02859.x 11967076

[48] Jia W Tovell N Clegg S Trimmer M Cole J A single channel for nitrate uptake, nitrite export and nitrite uptake by *Escherichia coli* NarU and a role for NirC in nitrite export and uptake. Biochem J 2009; 417:297-304 10.1042/BJ20080746 18691156

[49] Zumft WG Cell biology and molecular basis of denitrification. Microbiol Mol Biol Rev 1997; 61:533-616 9409151 10.1128/mmbr.61.4.533-616.1997 PMC232623

[50] Adman ET Godden JW Turley S The structure of copper-nitrite reductase from *Achromobacter cycloclastes* at five pH values, with NO_2_^-^ bound and with type II copper depleted. J Biol Chem 1995; 270:27458-27474 10.1074/jbc.270.46.27458 7499203

[51] Edgar RC MUSCLE: multiple sequence alignment with high accuracy and high throughput. Nucleic Acids Res 2004; 32:1792-1797 10.1093/nar/gkh340 15034147 PMC390337

[52] Fields BA Guss JM Freeman HC Three-dimensional model for stellacyanin, a "blue" copper-protein. J Mol Biol 1991; 222:1053-1065 10.1016/0022-2836(91)90593-U 1762145

[53] Hart PJ Nersissian AM Herrmann RG Nalbandyan RM Valentine JS Eisenberg D A missing link in cupredoxins: crystal structure of cucumber stellacyanin at 1.6 Å resolution. Protein Sci 1996; 5:2175-2183 10.1002/pro.5560051104 8931136 PMC2143285

[54] Romero A Hoitink CW Nar H Huber R Messerschmidt A Canters GW X-ray analysis and spectroscopic characterization of M121Q azurin. A copper site model for stellacyanin. J Mol Biol 1993; 229:1007-1021 10.1006/jmbi.1993.1101 8383207

[55] Hinchliffe P Carroll J Sazanov LA Identification of a novel subunit of respiratory complex I from *Thermus thermophilus.* Biochemistry 2006; 45:4413-4420 10.1021/bi0600998 16584177

[56] Simon J Gross R Einsle O Kroneck PM Kröger A Klimmek O A NapC/NirT-type cytochrome c (NrfH) is the mediator between the quinone pool and the cytochrome c nitrite reductase of *Wolinella succinogenes.* Mol Microbiol 2000; 35:686-696 10.1046/j.1365-2958.2000.01742.x 10672190

[57] Mooser D Maneg O Corvey C Steiner T Malatesta F Karas M Soulimane T Ludwig B A four-subunit cytochrome *bc*_1_ complex complements the respiratory chain of *Thermus thermophilus.* Biochim Biophys Acta 2005; 1708:262-274 10.1016/j.bbabio.2005.03.008 15869739

[58] Olendzenski L Liu L Zhaxybayeva O Murphey R Shin DG Gogarten JP Horizontal transfer of archaeal genes into the *Deinococcaceae*: detection by molecular and computer-based approaches. J Mol Evol 2000; 51:587-599 11116332 10.1007/s002390010122

[59] Kieft TL Fredrickson JK Onstott TC Gorby YA Kostandarithes HM Bailey TJ Kennedy DW Li SW Plymale AE Spadoni CM Gray MS Dissimilatory reduction of Fe(III) and other electron acceptors by a *Thermus* isolate. Appl Environ Microbiol 1999; 65:1214-1221 10049886 10.1128/aem.65.3.1214-1221.1999 PMC91167

[60] Richter K Schicklberger M Gescher J Dissimilatory reduction of extracellular electron acceptors in anaerobic respiration. Appl Environ Microbiol 2012; 78:913-921 10.1128/AEM.06803-11 22179232 PMC3273014

[61] Friedrich CG Bardischewsky F Rother D Quentmeier A Fischer J Prokaryotic sulfur oxidation. Curr Opin Microbiol 2005; 8:253-259 10.1016/j.mib.2005.04.005 15939347

[62] Skirnisdottir S Hreggvidsson GO Holst O Kristjansson JK Isolation and characterization of a mixotrophic sulfur-oxidizing *Thermus scotoductus.* Extremophiles 2001; 5:45-51 10.1007/s007920000172 11302502

[63] Friedrich CG Rother D Bardischewsky F Quentmeier A Fischer J Oxidation of reduced inorganic sulfur compounds by bacteria: emergence of a common mechanism? Appl Environ Microbiol 2001; 67:2873-2882 10.1128/AEM.67.7.2873-2882.2001 11425697 PMC92956

[64] Jormakka M Yokoyama K Yano T Tamakoshi M Akimoto S Shimamura T Curmi P Iwata S Molecular mechanism of energy conservation in polysulfide respiration. Nat Struct Mol Biol 2008; 15:730-737 10.1038/nsmb.1434 18536726 PMC2887006

[65] Krafft T Gross R Kröger A The function of *Wolinella succinogenes psr* genes in electron transport with polysulphide as the terminal electron acceptor. Eur J Biochem 1995; 230:601-606 10.1111/j.1432-1033.1995.0601h.x 7607234

[66] Glaser P Danchin A Kunst F Zuber P Nakano MM Indentification and isolation of a gene required for nitrate assimilation and anaerobic growth of *Bacillus subtilis.* J Bacteriol 1995; 177:1112-1115 7860592 10.1128/jb.177.4.1112-1115.1995 PMC176711

[67] Aravind L Tatusov RL Wolf YI Walker DR Koonin EV Evidence for massive gene exchange between archaeal and bacterial hyperthermophiles. Trends Genet 1998; 14:442-444 10.1016/S0168-9525(98)01553-4 9825671

[68] Dodsworth JA Li L Wei S Hedlund BP Leigh JA de Figueiredo P Interdomain conjugal transfer of DNA from bacteria to archaea. Appl Environ Microbiol 2010; 76:5644-5647 10.1128/AEM.00967-10 20581182 PMC2918978

[69] Schwarzenlander C Averhoff B Characterization of DNA transport in the thermophilic bacterium *Thermus thermophilus* HB27. FEBS J 2006; 273:4210-4218 10.1111/j.1742-4658.2006.05416.x 16939619

[70] Schwarzenlander C Haase W Averhoff B The role of single subunits of the DNA transport machinery of *Thermus thermophilus* HB27 in DNA binding and transport. Environ Microbiol 2009; 11:801-808 10.1111/j.1462-2920.2008.01801.x 19396940

[71] Markowitz VM Mavromatis K Ivanova NN Chen IM Chu K Kyrpides NC IMG ER: a system for microbial genome annotation expert review and curation. Bioinformatics 2009; 25:2271-2278 10.1093/bioinformatics/btp393 19561336

[72] Averhoff B Shuffling genes around in hot environments: the unique DNA transporter of *Thermus thermophilus.* FEMS Microbiol Rev 2009; 33:611-626 10.1111/j.1574-6976.2008.00160.x 19207744

[73] Cava F Zafra O da Costa MS Berenguer J The role of the nitrate respiration element of *Thermus thermophilus* in the control and activity of the denitrification apparatus. Environ Microbiol 2008; 10:522-533 10.1111/j.1462-2920.2007.01472.x 18199125

[74] Dodsworth JA Hungate BA Hedlund BP Ammonia oxidation, denitrification and dissimilatory nitrate reduction to ammonium in two US Great Basin hot springs with abundant ammonia-oxidizing archaea. Environ Microbiol 2011; 13:2371-2386 10.1111/j.1462-2920.2011.02508.x 21631688

